# Motion of the rearfoot, ankle and subtalar joints and ankle moments when wearing lateral wedge insoles – results from bone anchored markers

**DOI:** 10.1186/1757-1146-5-S1-O10

**Published:** 2012-04-10

**Authors:** Richard Jones, Chris Nester, Anmin Liu, Peter Wolf, Anton Arndt, Paul Lundgren, Arne Lundberg

**Affiliations:** 1 Centre for Health Sciences Research, University of Salford, Salford, Greater Manchester, M6 6PU, UK; 2 Sensory Motor Laboratory, ETH Zurich Switzerland; 3 Karolinska Hospital , Huddinge, Sweden

## Background

Knee osteoarthritis is a debilitating condition and increased dynamic loading at the knee has been linked with increased progression of the disease. Lateral wedge insoles have been used in clinical practice since the late 1980s. It is theorised that lateral wedge insoles increase the subtalar joint valgus orientation and increase the ankle valgus moment [1], with subsequent reduced knee varus moments [2]. Results have shown that both clinical success and reductions in knee loading vary between people. Differences could be due to person specific foot biomechanics. The aim of this study was to determine the changes in frontal plane foot and ankle motion and moment due to a lateral wedge orthosis.

## Materials and methods

Five healthy individuals participated in the study (age, height). An intracortical pin approach to measurement of foot motion was adopted because it enables effects of the orthosis at the individual ankle and subtalar joints to be identified. Accordingly, 1.2 mm intracortical pins were inserted into 9 bones of the foot and leg under local anaesthesia and reflective markers attached to the pins. Individuals walked over a force platform ten times in a control shoe condition and the Salford Lateral Wedge Technology insole™. All data were normalised to stance phase. Orthotic effect was evaluated using motions between tibia-talus, talus-calcaneus, and tibia-calcaneus during the first 50% of stance phase.

## Results

**Table 1:** Raw changes in three segments (-ve change degs = increased eversion) and ankle moment (negative change = increased eversion moment nm/kg) in the 5 individuals compared to control condition.

	S1	S2	S3	S4	S5
Tibia-talus y	-2.91	-1.18	-1.71	-1.14	-3.81
Talo-calcaneal y	-4.27	-1.96	-6.04	-2.34	-2.94
Tibia-calcaneal	-1.19	-2.15	-4.54	-1.90	-1.19
Ankle joint coronal moment	-0.09	-0.02	XX	-0.05	-0.06

## Conclusions

Although the lateral wedge insole offers a change in the ankle valgus moment, each person’s kinematic response varied (Table 1). This demonstrates that the motions do not occur at one segment as per previous research [1] and therefore other motions in adjacent joints needs to be considered. This is potentially one of the reasons why some people do not respond to the orthosis and suggests that use of biomechanical foot classifications could enable more targeted use of lateral wedge insoles [3].
